# Loss of *dmrt2a* Alters Hepatic LC-PUFA Composition and Lipid Homeostasis in Zebrafish

**DOI:** 10.3390/cells15191763

**Published:** 2026-09-27

**Authors:** Mingxi Hou, Donghao Li, Yaqun Zhang, Hengde Li

**Affiliations:** 1 Key Laboratory of Aquatic Genomics, Ministry of Agriculture and Rural Affairs, Beijing Key Laboratory of Fishery Biotechnology, Chinese Academy of Fishery Sciences, Beijing 100141, China; ldh202403@163.com (D.L.); zhangyq@cafs.ac.cn (Y.Z.); 2 National Demonstration Center for Experimental Fisheries Science Education, Shanghai Ocean University, Shanghai 201306, China

**Keywords:** *dmrt2a*, LC-PUFA, DHA, lipid metabolism, hepatic steatosis

## Abstract

Long-chain polyunsaturated fatty acids (LC-PUFAs), particularly eicosapentaenoic acid (EPA) and docosahexaenoic acid (DHA), are essential nutrients for human health, and fish and aquatic products are the primary dietary sources of these bioactive lipids. The endogenous capacity of teleosts to synthesize LC-PUFAs directly determines their nutritional value for human consumers. However, the genetic regulators governing this biosynthetic pathway remain largely unexplored. Here, we show that targeted disruption of *dmrt2a*, a transcription factor gene, in zebrafish (*Danio rerio*) via CRISPR/Cas9, alters hepatic LC-PUFA composition and lipid homeostasis. Homozygous *dmrt2a*^−/−^ mutants exhibited significantly reduced molar percentages of EPA (C20:5n-3) and DHA (C22:6n-3) in the liver, accompanied by the accumulation of their precursors, C18:2n-6 and C18:3n-3. In addition, the mutants developed a hepatic lipid metabolic disorder characterized by pathological accumulation of lipid droplets and elevated triglyceride levels, thereby providing a novel zebrafish model for research on hepatic steatosis. Transcriptomic profiling revealed that differentially expressed genes between *dmrt2a*^−/−^ mutant and wild-type livers were significantly enriched in fatty acid biosynthesis and metabolic pathways. Notably, an LC-PUFA-enriched diet partially rescued the aberrant hepatic lipid phenotype. To our knowledge, this is the first study in any fish species to link a DMRT-family transcription factor to hepatic LC-PUFA composition and lipid homeostasis, suggesting *dmrt2a* as a potential target for genetic breeding to improve the nutritional quality of aquaculture products.

## 1. Introduction

Long-chain polyunsaturated fatty acids (LC-PUFAs), particularly eicosapentaenoic acid (EPA, 20:5n-3) and docosahexaenoic acid (DHA, 22:6n-3), are essential nutrients for human health [1,2]. These fatty acids serve not only as structural components of cell membranes, but also as signaling molecules and ligands for nuclear receptors, thereby regulating a range of physiological processes, including lipid metabolism, oxidative stress, and inflammation [3]. Fish and fishery products represent the primary dietary source of these LC-PUFAs [4]. The endogenous capacity for LC-PUFA synthesis in fish directly affects their nutritional value as human food [5].

The liver is the primary site of lipid synthesis in fish, accounting for approximately 90% of total lipid production, and plays a crucial role in maintaining metabolic homeostasis among carbohydrates, amino acids, and fatty acids [6]. The synthesis of endogenous LC-PUFAs depends on desaturases and elongases [7]; however, the regulatory mechanisms governing these enzymes remain poorly understood. Thus, elucidating the genetic regulation of LC-PUFA biosynthesis in commercially important fish species is of considerable importance for both food science and the aquaculture industry.

Non-alcoholic fatty liver disease (NAFLD) is a highly prevalent metabolic liver disease worldwide [8]. Although the pathogenesis of NAFLD remains incompletely understood, hepatic lipid metabolic disorders, particularly aberrant triglyceride accumulation and lipid droplet formation, are widely regarded as core events driving the initiation and progression of this disease [9,10]. Insufficient dietary intake or impaired endogenous synthesis of LC-PUFAs can promote the development and progression of NAFLD [11]. Therefore, deciphering the key genes governing endogenous LC-PUFA biosynthesis not only helps elucidate the regulatory network of lipid metabolism in fish, but also provides a foundation for developing animal models to study hepatic steatosis.

The DMRT (doublesex and mab-3 related transcription factor) family is characterized by a highly conserved N-terminal DM domain that confers DNA-binding activity and a less conserved C-terminal functional domain [12,13,14]. DMRT genes are homologous to doublesex (*dsx*) in *Drosophila melanogaster* and male abnormal 3 (*mab-3*) in *Caenorhabditis elegans* [15,16]. They encode transcription factors involved in sex determination and embryonic development [17,18].

In recent years, the functional repertoire of DMRT family members has expanded considerably [19]. In teleosts, *dmrt2* is also designated as *dmrt2a* or *terra*, and the teleost-specific *dmrt2b* originated from *dmrt2* during the teleost-specific genome duplication event approximately 350 million years ago [20]. Previous studies have shown that *dmrt2a* is involved in key processes such as somite symmetry establishment, left–right axis patterning, and fast muscle differentiation in zebrafish; knockdown of *dmrt2a* results in embryonic developmental abnormalities [15,21]. However, its role in regulating LC-PUFA synthesis and hepatic lipid homeostasis remains unexplored.

Gene editing technologies have been widely shown to profoundly alter fatty acid metabolism in fish, opening new avenues for genetic improvement of the nutritional quality of aquatic products. In zebrafish (*Danio rerio*), knockout of *elovl4b* markedly reduces the biosynthetic capacity for very-long-chain PUFAs [22], and CRISPR/Cas9-mediated disruption of *ehd3* significantly increases muscle levels of EPA, DHA, and total n-3 PUFAs without affecting growth performance [23]. In common carp (*Cyprinus carpio*), overexpression of *fat1* up-regulates elovl5 expression by approximately 6.5-fold, thereby modulating the PUFA elongation pathway [24].

To investigate the role of *dmrt2a*, we generated *dmrt2a* knockout zebrafish using CRISPR/Cas9. We performed multi-dimensional comparisons between wild-type and *dmrt2a^−/−^* fish, including hepatic LC-PUFA compositional profiling, assessment of hepatic lipid accumulation, quantification of hepatic triglyceride contents, and liver transcriptome analysis. We further conducted a dietary intervention experiment to examine whether LC-PUFA-enriched feeding could ameliorate the aberrant hepatic lipid phenotype induced by *dmrt2a* loss. This study aimed to elucidate, for the first time, the functional association between *dmrt2a* and the regulation of hepatic lipid metabolism in zebrafish.

## 2. Materials and Methods

### 2.1. Animal Maintenance

Zebrafish (AB strain) were maintained in an indoor recirculating system at 28 °C under a 14 h light/10 h dark cycle, following standard zebrafish husbandry protocols [25]. Wild-type (WT) and *dmrt2a*^−/−^ mutant zebrafish were co-housed in the same tank from the fertilized egg stage until they were genotyped at adulthood (4 months of age) for experimental procedures. Stocking densities were 50 larvae/L at 5–15 dpf (days post-fertilization), 10–20 fish/L from 15 dpf to 2 mpf, and 3–6 fish/L from 2 mpf until 4 mpf; thereafter, adult fish were housed in 3 L tanks at approximately 1–2 fish/L (3–6 fish per tank). The recirculating water was maintained at pH 7.0–7.5, and dissolved oxygen was maintained at ≥ 6.0 mg/L. All fish were fed daily with *Artemia* containing trace amounts of LC-PUFA.

For the rescue experiment, 4-month-old WT and *dmrt2a*^−/−^ mutant zebrafish were acclimated for 3 days on the standard diet. After acclimation, fish received either the standard diet or an LC-PUFA-enriched diet for one month. Three groups were established: WT fed the standard diet, *dmrt2a*^−/−^ mutants fed the standard diet, and *dmrt2a*^−/−^ mutants fed the LC-PUFA-enriched diet. Each group contained three fish, each serving as one biological replicate. Throughout the experimental period, fish were fed twice daily at a total daily ration of 5% of body weight, with uneaten feed removed 10 min after each feeding. At the end of the feeding trial, all three fish in each group were euthanized with MS-222 (40 mg/L), and the liver of each fish was collected and analyzed. The composition of the standard diet is provided in Table S1. The LC-PUFA-enriched diet was prepared by supplementing the standard diet with 30 g fish oil per kg, and its fatty acid profile is shown in Table S2.

All animal experiments were approved by the Animal Care and Use Committee of the Chinese Academy of Fishery Sciences and were conducted in accordance with the principles of animal care and use for scientific purposes.

### 2.2. Generation and Identification of dmrt2a Knockout Fish

Genetic mutants were generated using a CRISPR/Cas9-mediated knockout strategy. The guide RNA (gRNA) targeting *dmrt2a* was designed using the online tool CRISPRscan (https://www.crisprscan.org/sequence/). The in vitro transcription template was amplified from the pT7-gRNA plasmid [26]. The T7 High Yield RNA Transcription Kit (Vazyme, Nanjing, China) was used to synthesize the gRNA, which was subsequently purified with VAHTS RNA Clean Beads (Vazyme, Nanjing, China) according to the manufacturer’s instructions. The gRNA concentration was measured using a NanoDrop One spectrophotometer (Thermo Fisher Scientific, Waltham, MA, USA). The gRNA (150 pg/embryo) was co-injected with TrueCut™ Cas9 Protein v2 (500 pg/embryo) (Thermo Fisher Scientific, USA) into one-cell-stage fertilized eggs to generate F0 mutants. F0 mutants were backcrossed to wild-type fish to obtain heterozygous F1 progeny. F1 heterozygotes carrying the same mutation were intercrossed to yield F2 offspring. F2 fish were reared to adulthood and genotyped by Sanger sequencing of PCR products covering the target locus to identify WT, heterozygous (Hetero), and homozygous (Homo) individuals for subsequent phenotypic analysis. We generated two homozygous mutant lines carrying 4 bp and 14 bp deletions, respectively. Both lines were analyzed independently for hepatic phenotype, while all other experiments were performed using the 4 bp deletion line. Primers used in this study are listed in Table S3.

### 2.3. RNA Isolation and Real-Time Quantitative PCR (RT-qPCR)

Total RNA was isolated from the liver tissues using TRIzol reagent (Invitrogen, Carlsbad, CA, USA) according to the manufacturer’s protocol. RNA integrity was assessed by agarose gel electrophoresis, and its concentration and purity were quantified using a Nanodrop One spectrophotometer (Thermo Fisher Scientific, USA). To eliminate potential genomic DNA contamination, the isolated RNA was treated with RNase-free DNase I (Promega, Madison, WI, USA). cDNA was synthesized by reverse transcription of the purified total RNA using the Rever Tra-AceM-MLV kit (TOYOBO, Tokyo, Japan). The cDNA product was diluted 5-fold and used as a template for real-time quantitative PCR (RT-qPCR) on a Bio-Rad CFX96Touch Detection System (Bio-Rad, Hercules, CA, USA). Each reaction mixture consisted of 5 μL 2 × Taq Pro Universal SYBR qPCR Master Mix (Vazyme, China), 3.5 μL dd H2O, 0.25 μL forward and reverse primers, and 1 μL diluted cDNA. The thermal cycling program was as follows: 95 °C for 3 min, followed by 45 cycles at 95 °C for 15 s, 60 °C for 15 s, and 72 °C for 15 s. Each sample was analyzed in three technical replicates, and the mean Ct value was used for quantification. Melt-curve analysis was performed for each reaction, and each instrument run included a no-template control and a no-reverse-transcriptase control for each primer pair. The *β-actin* was selected as a reference gene. Relative mRNA expression levels were calculated using the 2^−ΔCt^ or 2^−ΔΔCt^ method. Primers used for RT-qPCR and their amplification efficiencies (97–101%, R^2^ > 0.99) are listed in Table S3.

### 2.4. Assessment of Hepatic Lipid Content

Oil Red O (ORO) staining was performed on liver sections to visualize neutral lipids and assess lipid droplet accumulation. Liver tissues were fixed overnight in 4% paraformaldehyde in PBS, then dehydrated in 30% sucrose at 4 °C. The tissues were then embedded in Tissue Freezing Medium (Leica Biosystems, Germany) and frozen by floating in an ethanol–dry ice slurry. Sections (10 μm thick) were cut using a cryostat (Leica CM1950, Wetzlar, Germany) at –18 °C. Sections were incubated with the ORO working solution for 2 min in accordance with the instructions provided with the Modified Oil Red O Staining Kit (YEASEN, Shanghai, China). Sections were counterstained with hematoxylin to visualize nuclei. Images were acquired using an automatic digital slide scanner, Aperio Versa 8 (Leica, Germany). Six random fields per experimental group were captured for image analysis and quantification. Each field originated from a separate liver section of a distinct fish, with one field per individual and six fish per group. Lipid accumulation in the liver was quantified based on pixel counts using ImageJ software (v1.53k) [27].

Liver triglyceride concentrations were determined using a commercial triglyceride assay kit (Nanjing Jiancheng Bioengineering Institute, Nanjing, China), according to the manufacturer’s instructions. Briefly, liver samples (approximately 10 mg each) were weighed and homogenized in nine volumes (*w*/*v*) of phosphate-buffered saline (PBS, 0.1 M, pH 7.4) at 4 °C. The homogenates were centrifuged at 2500 rpm for 10 min, and 10 μL of the supernatant was mixed with 1000 μL of the working solution and incubated at 37 °C for 10 min. Glycerol standards were used for calibration. Total protein concentration in the supernatant was determined using a BCA protein assay kit (Nanjing Jiancheng Bioengineering Institute, Nanjing, China). Triglyceride content was normalized to total protein concentration. Each sample was assayed in triplicate, and six fish per group were analyzed.

### 2.5. RNA Sequencing and Data Analysis

To compare the transcriptomic profiles between the knockout and wild-type groups, three biological replicates per group were generated, with each replicate consisting of pooled liver tissues from three individual fish. RNA libraries were constructed according to a previously described protocol [28]. The libraries were sequenced on the Illumina NovaSeq 6000 platform, generating 150 bp paired-end reads. Raw reads were processed using fastp (v0.23.1) to remove adapter sequences, poly-N reads, and low-quality reads. Clean reads were then aligned to the GRCz11 reference genome [29] using HISAT2 (v2.2.1). Gene expression levels were quantified and normalized as fragments per kilobase of transcript per million mapped reads (FPKM). Differential expression analysis was performed using the DESeq2 R package (v1.42.0) with raw counts as input [30]. The Benjamini–Hochberg method was applied to adjust *p*-values for multiple testing; candidate genes with adjusted *p*-values < 0.05 were considered statistically significant. Differentially expressed genes were then subjected to functional enrichment analysis using the ClusterProfiler R package (v3.9) for Gene Ontology (GO) and Kyoto Encyclopedia of Genes and Genomes (KEGG) pathway enrichment [31]. Given the limited number of enriched terms obtained in the present study, terms with a raw *p*-value < 0.05 were selected for subsequent exploratory functional interpretation.

### 2.6. Determination of Fatty Acid Composition

Fatty acid composition was determined using gas chromatography coupled with a flame-ionization detector. Lipids were extracted from tissues by overnight treatment with chloroform/methanol (2:1, *v*/*v*) containing 0.005% butylated hydroxytoluene [32]. After extraction, the solvent was evaporated, and the total lipid extract was weighed. The tissue lipid content was calculated as the ratio of total lipid weight to the initial tissue weight. Exactly 0.2 mg of extracted lipid was methylated by incubation with 5% H_2_SO_4_ in methanol (*v*/*v*) at 100 °C for 1 h, and the resulting fatty acid methyl esters (FAMEs) were extracted with hexane and adjusted to a final volume of 3 mL. FAMEs were analyzed using an Agilent 7890A GC system (Agilent Technologies, Santa Clara, CA, USA) equipped with a flame ionization detector and an Agilent DB-23 capillary column (Agilent Technologies, USA). The oven temperature was programmed as follows: initially held at 60 °C for 1 min, then increased to 150 °C at 10 °C/min, to 200 °C at 15 °C/min, and finally to 230 °C at 30 °C/min, after which it was maintained for 5 min. The injector and detector temperatures were both set at 300 °C. The type of each FA was determined by comparing the retention time of the samples with the peaks of a Supelco 37-component FAME standard combination (Nu-Chek Prep, Inc., Elysian, MN, USA). Calibration curves were constructed by injecting the Supelco 37-component FAME standard at different concentrations under the same GC conditions. The absolute content of each FA was calculated using the external standard method based on the calibration curves and the conversion coefficient. The mol% of each fatty acid was calculated by dividing its free fatty acid mass by its molecular weight and normalizing to the sum of molar amounts of all detected fatty acids.

### 2.7. Statistical Analyses

All data are presented as the mean ± standard error of the mean (SEM). Statistical analyses were performed using SPSS 18.0. Normality was assessed using the Shapiro–Wilk test, and homogeneity of variances was assessed using Levene’s test; all data in the present study met these assumptions. For two-group comparisons, an unpaired, two-tailed Student’s *t*-test was used. For three-group comparisons, one-way ANOVA followed by Dunnett’s test was used, with the *dmrt2a*^−/−^ group as the reference. Statistical significance was defined as *p* < 0.05, *p* < 0.01, and *p* < 0.001, denoted by asterisks (*, **, ***, respectively). Graphical representations were generated with GraphPad Prism 10.1.2.

## 3. Results

### 3.1. Expression Profiling of dmrt2a in Zebrafish

We characterized the spatiotemporal expression pattern of *dmrt2a*. RT-qPCR analysis revealed that *dmrt2a* is a maternally expressed gene that is highly expressed during early embryogenesis and gradually decreases after the segmentation stage (Figure 1A). In adult zebrafish, *dmrt2a* was highly expressed in the gills and ovaries, with detectable levels in the liver and lower expression in other tissues, such as the heart and kidney (Figure 1B).

### 3.2. Generation of dmrt2a Mutant Lines

The zebrafish *dmrt2a* gene contains four exons. To elucidate its function, we employed CRISPR/Cas9-mediated gene editing, targeting the sequence 5’-GGCCCCGGAGAGGACGACACGGG-3’ (protospacer adjacent motif underlined), which is located within the second exon (Figure 2A). Following PCR amplification and Sanger sequencing, we identified two mutant alleles carrying a 4 bp deletion and a 14 bp deletion, respectively. Both deletions introduced a frameshift in the open reading frame, resulting in a truncated protein lacking the functional C-terminal domain (Figure 2B).

When heterozygous F1 mutants were intercrossed to produce F2 progeny, the genotype frequencies among adult survivors deviated from the expected 1:2:1 Mendelian ratio. Specifically, the ratio of WT to heterozygous individuals was approximately 1:2, whereas the number of homozygous mutants was markedly lower than expected, reaching only 30.36% of the WT count (Figure 2C,D). A chi-square goodness-of-fit test confirmed that the observed genotype distribution among adult survivors was significantly different from the expected Mendelian ratio (χ^2^ = 23.58, df = 2, *p* < 0.0001), suggesting a potential survival disadvantage for homozygous mutants before adulthood.

To further confirm the loss of function of *dmrt2a* in the mutants, we quantified *dmrt2a* mRNA levels by RT-qPCR. The results showed that the expression of *dmrt2a* was significantly reduced in the mutants relative to wild-type controls (*p* < 0.001) (Figure 2E). These results indicate that we successfully generated homozygous *dmrt2a* mutant lines.

### 3.3. dmrt2a Deletion Leads to Hepatic Lipid Metabolism Disorder

We unexpectedly observed that the livers of *dmrt2a*^−/−^ mutants appeared pathologically whitened and were prone to fragmentation during dissection, with extensive fat deposition in the abdominal cavity. This phenotype was observed in homozygous mutants from both the 4 bp and 14 bp deletion lines, and the 4 bp deletion line was used for all subsequent analyses. Oil Red O staining further revealed that lipid droplets were markedly more abundant in the livers of *dmrt2a*^−/−^ mutants (Figure 3A), with quantitative analysis showing an 88.6% increase in the ORO-positive area compared with wild-type controls (Figure 3B). Meanwhile, hepatic triglyceride levels in *dmrt2a*^−/−^ mutants were significantly higher than those in wild-type livers (Figure 3C). These results demonstrate that deletion of *dmrt2a* leads to aberrant hepatic lipid metabolism and excessive lipid accumulation in hepatocytes.

### 3.4. Transcriptomic Alterations in the Liver Following dmrt2a Deletion

To investigate the molecular mechanisms underlying the aberrant lipid metabolism in *dmrt2a*^−/−^ mutants, we performed RNA-sequencing of liver tissues from *dmrt2a*^−/−^ and wild-type fish. A total of 21.32 GB and 24.14 GB of raw data were generated from the knockout and wild-type groups, respectively. After quality control, clean reads were mapped to the reference genome; the total mapping rates were 90.19–90.55% in the WT group and 89.92–90.61% in the *dmrt2a*^−/−^ group. Detailed sequencing quality and mapping statistics are summarized in Table S4.

Principal component analysis (PCA) was performed to evaluate the overall transcriptomic variance and reproducibility of biological replicates. Biological replicates from each genotype clustered within their respective groups, and the WT group was clearly separated from the *dmrt2a*^−/−^ group (Figure 4A). These findings demonstrate good reproducibility of our RNA-seq data and indicate that *dmrt2a* deletion induces widespread transcriptional alterations. Differential expression analysis identified 77 upregulated and 117 downregulated genes in the liver following *dmrt2a* deletion (|log_2_FC| ≥ 1, adjusted *p* ≤ 0.05) (Figure 4B); these genes are listed in Table S5. Kyoto Encyclopedia of Genes and Genomes (KEGG) pathway enrichment showed that the differentially expressed genes were enriched in pathways related to fatty acid metabolism, including biosynthesis of unsaturated fatty acids, fatty acid elongation, and fatty acid degradation (Figure 4C). Gene Ontology (GO) analysis indicated that these differentially expressed genes were enriched in immune-related pathways, such as lymphocyte activation, leukocyte activation, and T cell activation involved in immune response (Figure 4D).

We analyzed the expression of key genes involved in fatty acid synthesis using RT-qPCR. The results revealed that *fads2* expression was significantly decreased, whereas *elovl8b* expression was increased. The expression levels of *elovl2*, *elovl4a*, *elovl4b*, *elovl5*, and *fasn* showed no significant difference between the knockout and wild-type groups (Figure 5). In addition, the expression of the fatty acid metabolism-related genes *rimkla*, *acot11b*, and *ptges3a* also differed significantly between *dmrt2a*^−/−^ mutants and wild-type controls (Figure 5).

### 3.5. Altered Hepatic LC-PUFA Composition in dmrt2a^−/−^ Fish

We further analyzed the changes in fatty acid composition in the livers of wild-type and *dmrt2a*^−/−^ mutant fish. The fatty acid profiles (mol%) are summarized in Table S6. Among the n-6 polyunsaturated fatty acids, the molar percentages of C18:3n-6 and C20:4n-6 in *dmrt2a*^−/−^ fish were significantly lower than those in the wild-type controls. In contrast, the molar percentages of their precursor C18:2n-6 were significantly increased (Figure 6A). Among the n-3 polyunsaturated fatty acids, the molar percentages of C20:5n-3 (EPA), C22:5n-3, and C22:6n-3 (DHA) were significantly decreased. In contrast, the molar percentages of their precursor C18:3n-3 were significantly elevated (Figure 6B). These results indicate that hepatic LC-PUFA composition, particularly EPA and DHA, is altered following *dmrt2a* deletion.

### 3.6. A LC-PUFA-Enriched Diet Alleviates Hepatic Steatosis in dmrt2a Mutants

Given that endogenous LC-PUFA synthesis is impaired in *dmrt2a*^−/−^ mutants, we fed *dmrt2a*^−/−^ mutants a LC-PUFA-enriched diet for one month. Feeding the LC-PUFA-enriched diet ameliorated the aberrant hepatic lipid metabolism in *dmrt2a*^−/−^ mutants (Figure 7A), with significant reductions in both lipid droplet density and hepatic triglyceride levels compared with mutants fed the standard diet (Figure 7B,C). However, neither parameter was restored to wild-type levels (Figure 7). Notably, the LC-PUFA-enriched diet was not strictly isolipidic or isoenergetic with the control diet, as fish oil supplementation increased total lipids and gross energy in addition to LC-PUFA. Since high-lipid or high-energy diets generally aggravate lipid accumulation, the reductions in lipid droplet accumulation and triglyceride levels are unlikely to be explained by increased total lipids or energy, but rather support a beneficial role of increased LC-PUFA supply.

## 4. Discussion

The *dmrt2a* is widely expressed in diverse tissues, suggesting its pleiotropic involvement in various biological processes. In zebrafish, morpholino-mediated knockdown of *dmrt2a* has been shown to induce severe developmental defects, including disrupted somite symmetry, aberrant left–right axis patterning, and impaired fast muscle differentiation [33,34]. In contrast, TALEN-mediated targeted disruption of *dmrt2a* produced homozygous mutants that exhibited only mild phenotypic abnormalities and remained viable to adulthood [35]. In the present study, homozygous *dmrt2a*^−/−^ mutants were present at a lower frequency than expected among adult survivors from heterozygous intercrosses, suggesting a potential survival disadvantage before adulthood. This may be partly attributable to maternal *dmrt2a* transcripts. Meanwhile, we observed aberrant hepatic lipid metabolism in surviving *dmrt2a*^−/−^ zebrafish, characterized by pathological liver paleness, accumulation of lipid droplets, and elevated triglyceride levels. These histological and biochemical features position the *dmrt2a* mutant as a valuable model for investigating the pathogenesis of hepatic steatosis. Given the established role of DMRT-family genes in sexual differentiation, we cannot exclude the possibility that sex-related differences contributed to the observed hepatic phenotypes.

Our further investigation revealed that the aberrant lipid metabolism caused by *dmrt2a* deficiency is associated with disruptions in endogenous LC-PUFA synthesis and metabolism. Transcriptomic analysis of livers showed that differentially expressed genes between the *dmrt2a*^−/−^ and wild-type groups were significantly enriched in fatty acid biosynthesis and metabolic pathways. LC-PUFAs have been reported to play a critical role in hepatic lipid metabolism [36,37]. Insufficient availability of LC-PUFAs disrupts hepatic lipid homeostasis and induces hepatic steatosis [38]. A higher dietary intake of n-3 LC-PUFAs, including EPA and DHA, is widely recognized to improve lipid metabolism, alleviate hepatic inflammation, and reduce hepatic steatosis [39,40].

The RT-qPCR results revealed significant down-regulation of *fads2*, which encodes a fatty acid desaturase critical for LC-PUFA biosynthesis [41,42,43]. Consistently, the levels of its substrates, C18:2n-6 and C18:3n-3, increased significantly. The altered *fads2* expression following *dmrt2a* deletion likely accounts for the impaired endogenous synthesis of EPA and DHA. However, the molecular mechanism by which *dmrt2a* regulates *fads2* remains unclear, and direct flux measurements are needed in future studies to confirm this impairment further. In addition, other differentially expressed genes related to lipid metabolism were identified, *acot11* modulates fatty acid oxidation and synthesis to maintain intracellular lipid homeostasis [44,45]; and *ptges3a* catalyzes the conversion of arachidonic acid (ARA, C20:4n-6) to prostaglandin E2 (PGE2) [46]. Given the dysregulated expression of these genes in *dmrt2a^−/−^* mutants, such alterations likely further contribute to the onset and progression of the hepatic lipid metabolism disorder.

LC-PUFAs play a critical role in maintaining hepatic lipid homeostasis [47,48,49]. They act as ligands for nuclear receptors, such as peroxisome proliferator-activated receptors (PPARs) and liver X receptors (LXRs), thereby regulating lipid synthesis and fatty acid oxidation [50,51,52]. Reduced hepatic levels of ARA and DHA trigger transcriptional activation of sterol regulatory element-binding protein 1c (SREBP-1c) and its downstream target genes involved in fatty acid and triglyceride synthesis, ultimately contributing to dysregulation of hepatic lipid metabolism [53]. Previous studies have shown that deficiency of *elovl2*, which encodes a key elongase essential for DHA biosynthesis, leads to enhanced lipogenesis, impaired lipid secretion, and subsequent hepatocyte injury [54,55]. Thus, we propose that impaired endogenous synthesis of EPA and DHA serves as the primary driver of hepatic lipid dysregulation following *dmrt2a* deletion. Moreover, immune-related pathways were concurrently enriched in the transcriptomic analysis of *dmrt2a*^−/−^ livers, suggesting a potential immune-associated transcriptional response [56,57]. However, further studies are needed to determine whether this reflects overt hepatic inflammation and whether it contributes to steatosis.

LC-PUFAs are essential nutrients for human health, and fish represent the primary dietary source of these fatty acids [58,59]. Enhancing the endogenous capacity to synthesize LC-PUFAs would improve their nutritional value for human consumers. From a food science perspective, our findings identify a previously unrecognized genetic factor that controls the intrinsic capacity for LC-PUFA synthesis. These findings provide a valuable reference for future efforts to improve the nutritional quality of aquaculture products. Nevertheless, these findings should be extrapolated to other fish species with caution. Although zebrafish is a well-established model, its small size and growth limitations make it not fully representative of aquaculture species. Future validation in commercially important fish species is needed.

## 5. Conclusions

In the present study, we demonstrate that targeted disruption of *dmrt2a* in zebrafish leads to altered hepatic LC-PUFA composition, particularly EPA and DHA, and concomitant hepatic lipid dysregulation (Figure 8). The molar percentages of EPA and DHA were significantly reduced in *dmrt2a^−/−^* mutants, whereas their precursors accumulated. These changes were accompanied by a significant down-regulation of *fads2*, which encodes a key desaturase in the LC-PUFA biosynthetic pathway. Moreover, dietary supplementation with a LC-PUFA-enriched diet partially ameliorated the aberrant hepatic lipid metabolism. To our knowledge, this is the first report to implicate a DMRT family transcription factor in the regulation of hepatic LC-PUFA status and lipid homeostasis in any fish species. These observations identify a zebrafish model conducive to research into hepatic steatosis and provide a theoretical foundation for genetic breeding to improve the nutritional quality of aquaculture products.

## Figures and Tables

**Figure 1:**
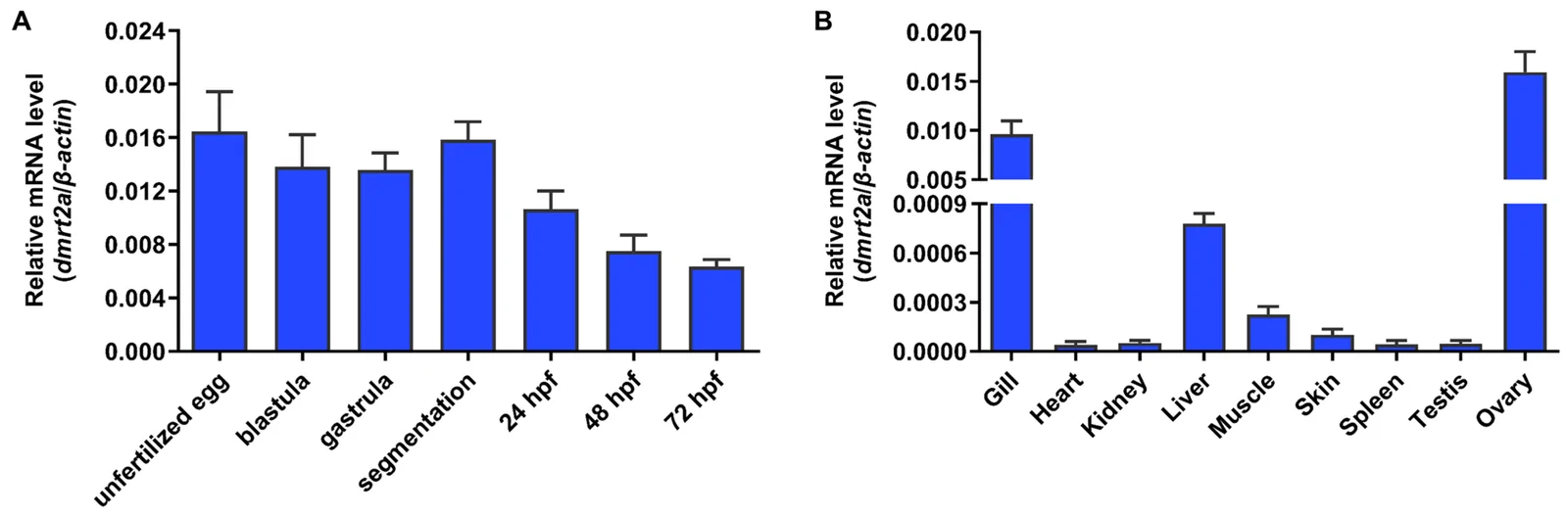
RT-qPCR analysis of the temporal and spatial expression patterns of *dmrt2a* in zebrafish. (**A**) Relative mRNA expression levels during embryonic development. (**B**) Relative mRNA expression levels in various adult tissues. Expression was normalized to *β-actin* using the 2^−ΔCt^ method; data are presented as mean ± SEM, *n* = 6; hpf, hours post-fertilization.

**Figure 2:**
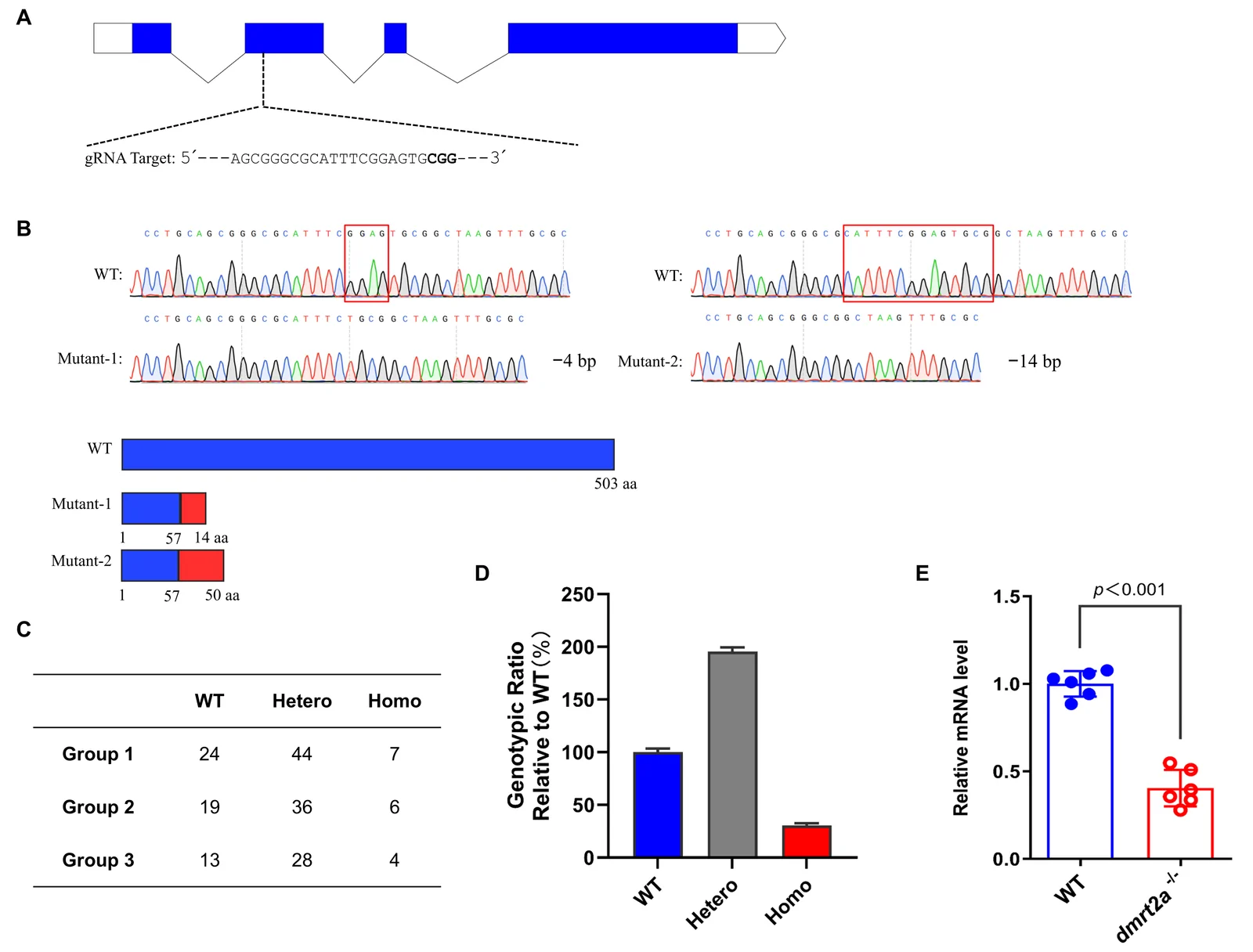
CRISPR/Cas9-mediated knockout of *dmrt2a* in zebrafish. (**A**) Schematic of *dmrt2a* genomic structure and gRNA targeting site. (**B**) Mutation locus and predicted protein truncation; a red box indicates deleted nucleotides. (**C**,**D**) Genotype counts (**C**) and percentages (**D**) of wild-type (WT), heterozygous (Hetero), and homozygous mutant (Homo) offspring derived from heterozygous intercrosses. (**E**) Relative *dmrt2a* mRNA expression in homozygous mutants normalized to that in WT; as determined by RT-qPCR using the 2^−ΔΔCt^ method; data are presented as mean ± SEM, *n* = 6.

**Figure 3:**
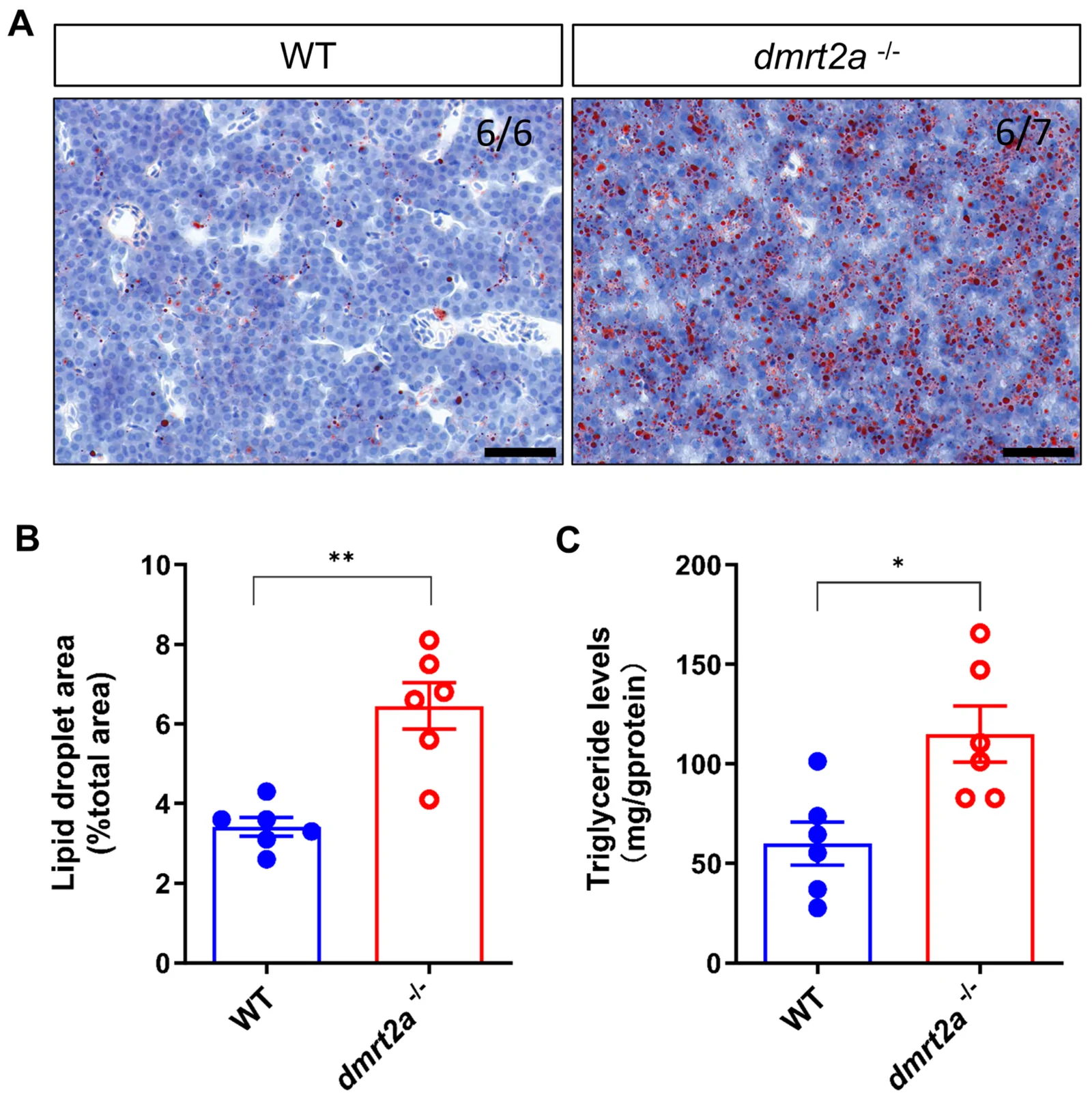
Hepatic lipid dysregulation in *dmrt2a*-deficient zebrafish. (**A**) Representative images of Oil Red O-stained liver sections from WT and mutant fish. Scale bar: 50 μm. (**B**) Quantitative analysis of Oil Red O staining intensity in the liver. (**C**) Triglyceride levels in WT and mutant livers. Data are presented as mean ± SEM, *n* = 6; * *p* < 0.05, ** *p* < 0.01.

**Figure 4:**
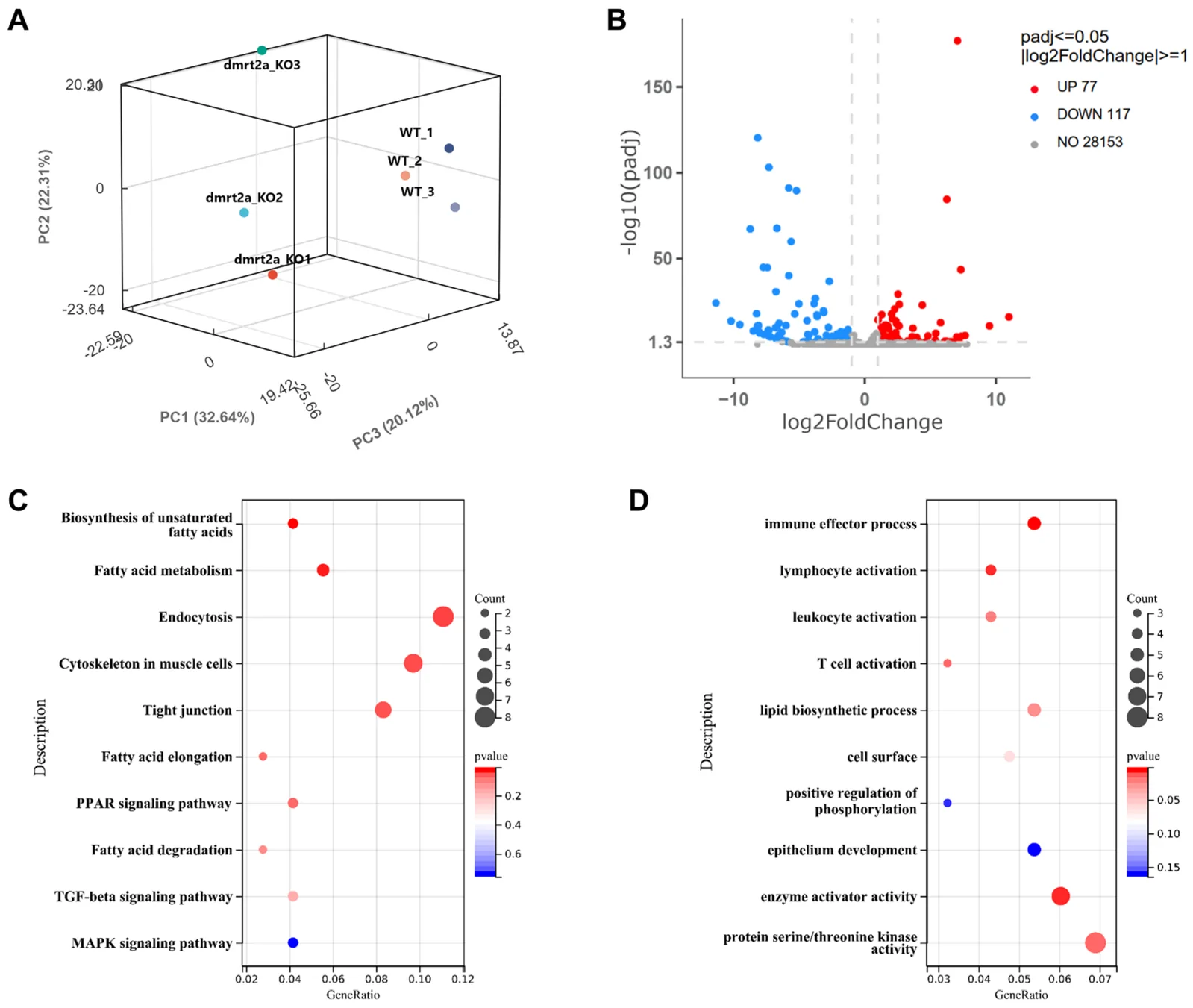
Transcriptome analysis of livers from WT and *dmrt2a* mutant zebrafish. (**A**) Three-dimensional PCA plot of transcriptomic profiles of WT and *dmrt2a*^−/−^ samples. (**B**) Volcano plot of differentially expressed genes. Red and blue dots represent upregulated and downregulated genes, respectively. (**C**) KEGG pathway enrichment analysis of differentially expressed genes. (**D**) GO enrichment analysis of differentially expressed genes. Bubble size represents the number of enriched genes, and the color gradient indicates the significance level. *n* = 3 biological replicates per genotype (3 fish pooled per replicate).

**Figure 5:**
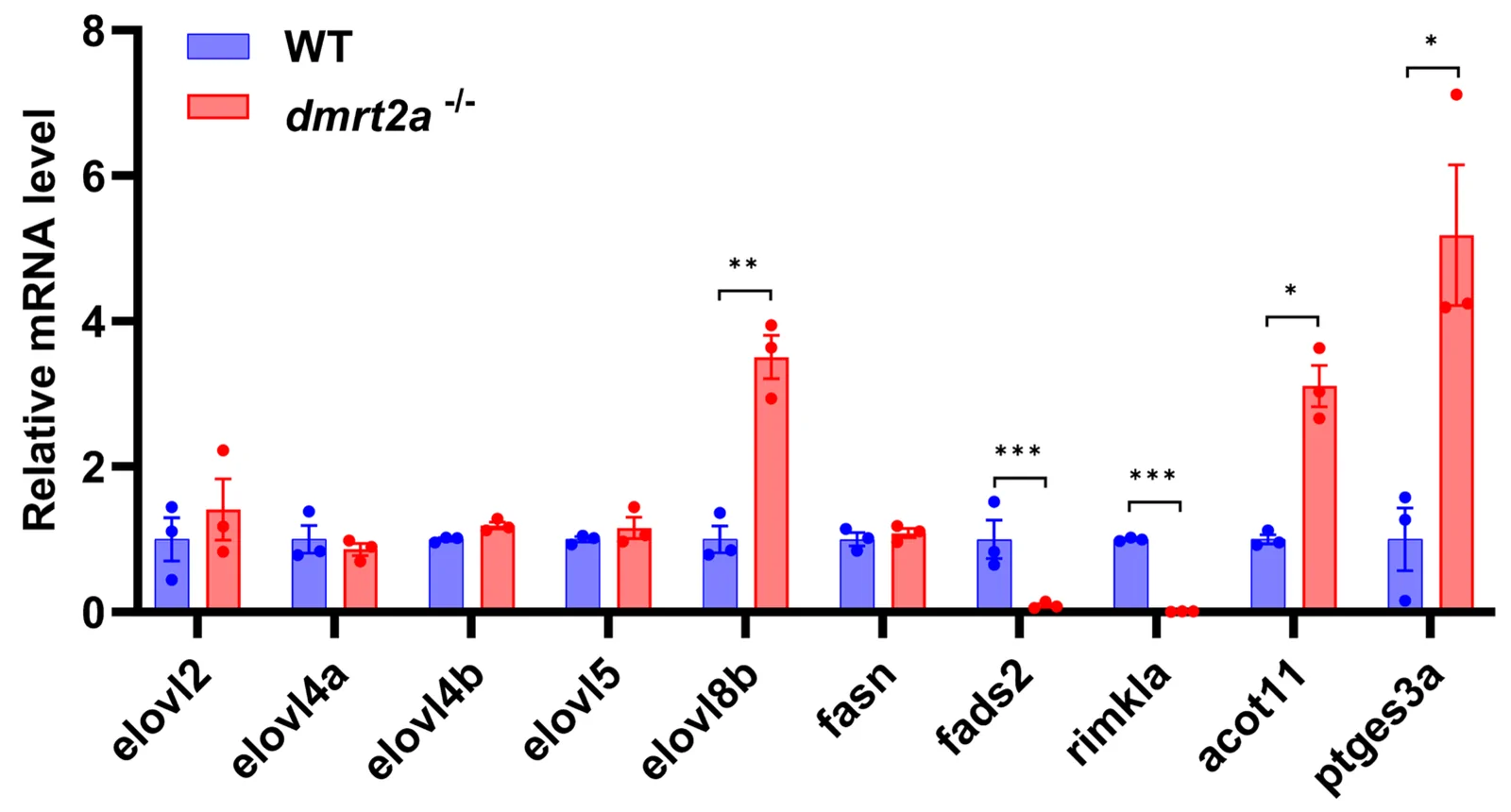
Relative mRNA levels of fatty acid metabolism genes in WT and mutant livers. Expression was normalized using the 2^−ΔΔCt^ method; data are presented as mean ± SEM, *n* = 3. * *p* < 0.05, ** *p* < 0.01, *** *p* < 0.001.

**Figure 6:**
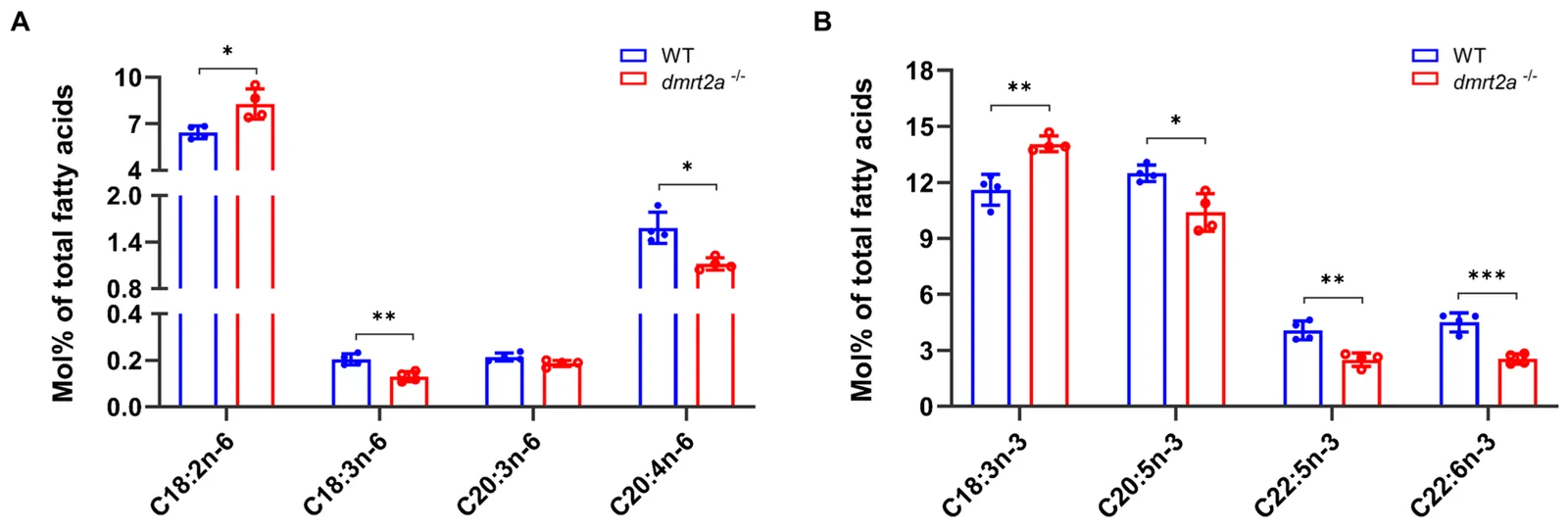
LC-PUFA composition in livers of WT and *dmrt2a* mutants. (**A**) Molar percentages of n-6 LC-PUFA. (**B**) Molar percentages of n-3 LC-PUFA. Data are presented as mean ± SEM, *n* = 4; Statistical significance was assessed using Student’s *t*-test; * *p* < 0.05, ** *p* < 0.01, *** *p* < 0.001 vs. WT.

**Figure 7:**
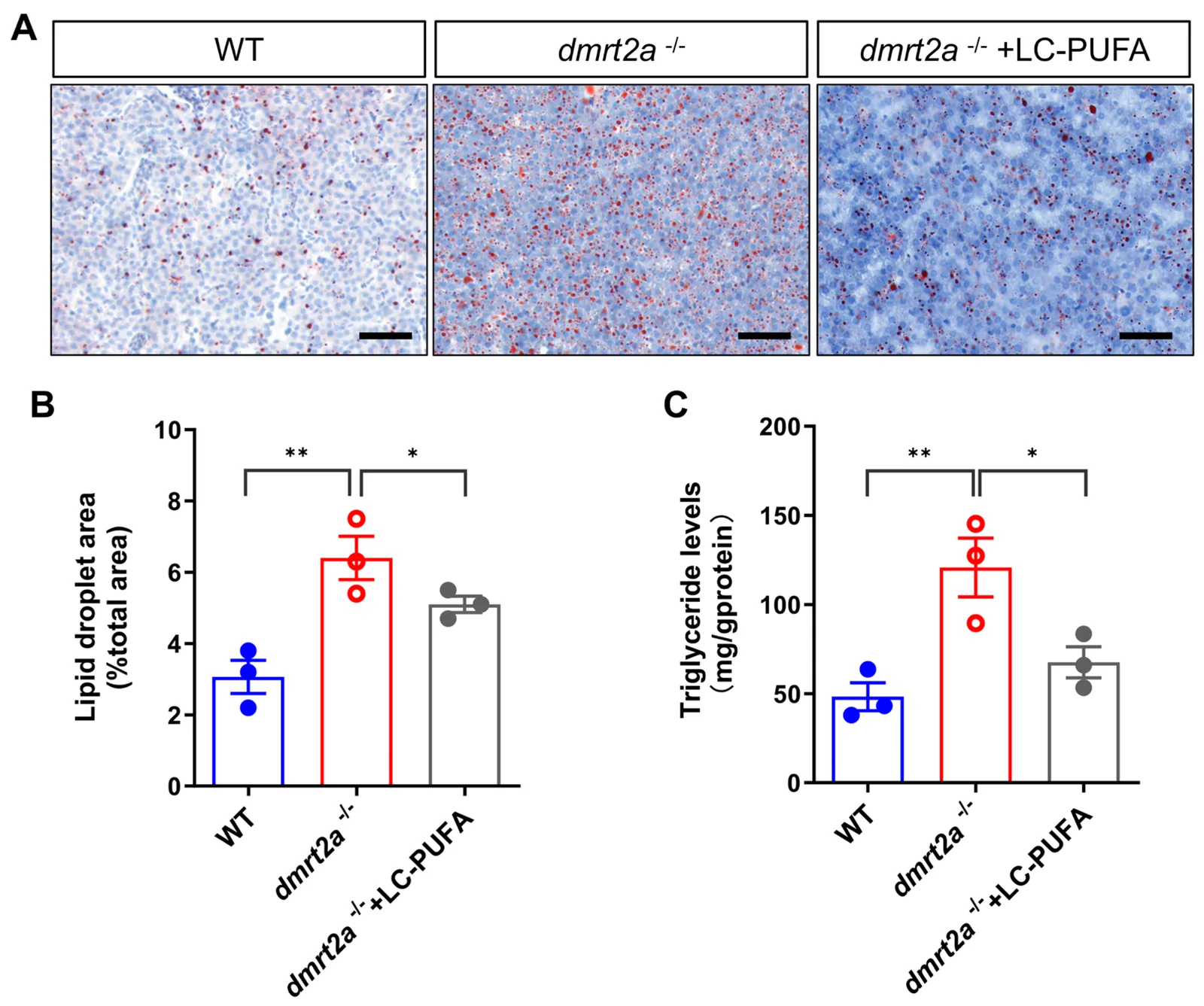
A LC-PUFA-rich diet rescues liver lipid dysregulation in *dmrt2a* mutant zebrafish. (**A**) Representative images of Oil Red O-stained liver sections from WT, *dmrt2a* mutants, and mutants fed a LC-PUFA-rich diet. Scale bar: 50 μm. (**B**) Quantification of Oil Red O staining intensity in the three groups. (**C**) Hepatic triglyceride levels. All data are presented as mean ± SEM, *n* = 3. * *p* < 0.05, ** *p* < 0.01.

**Figure 8:**
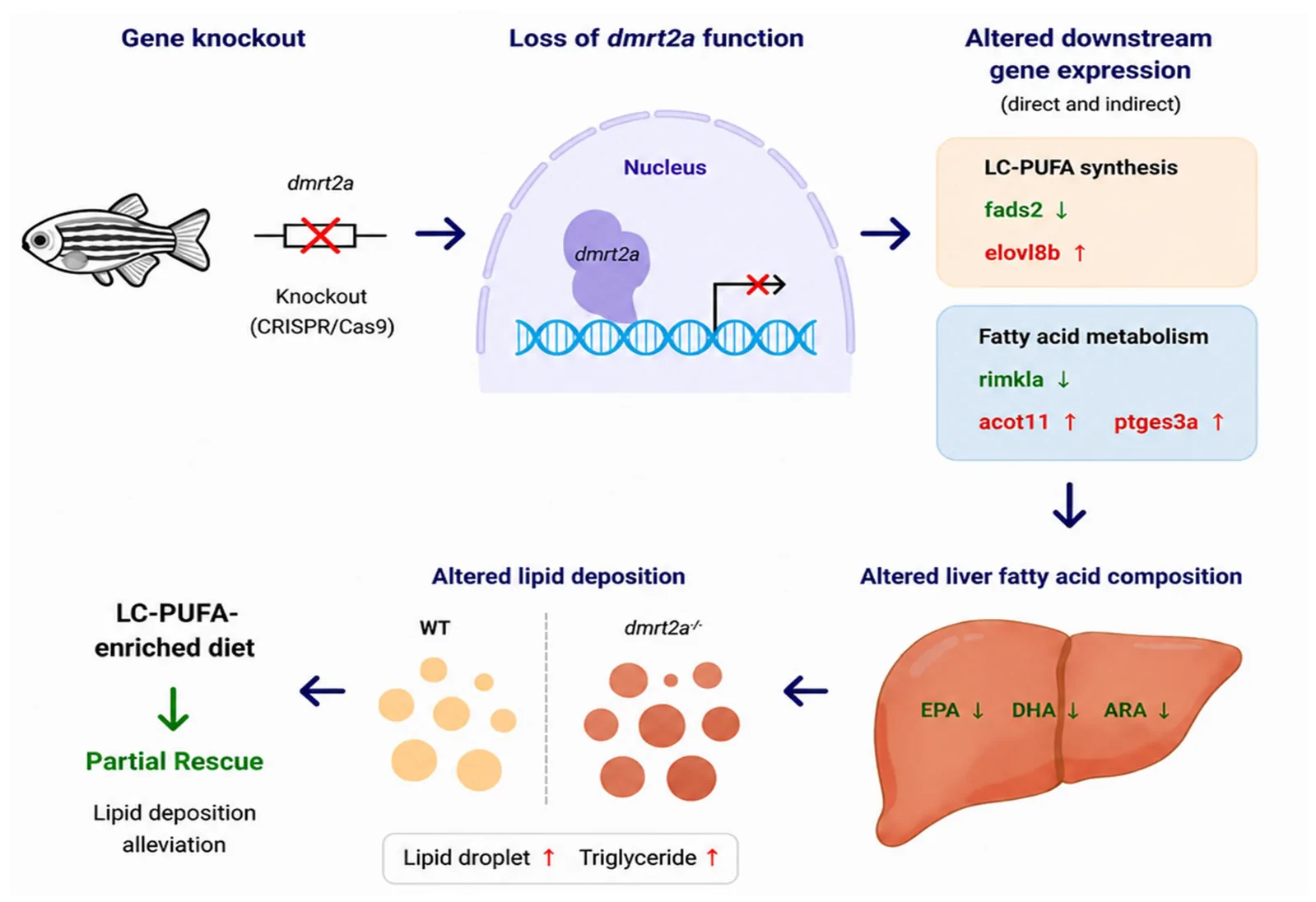
Proposed model for the role of *dmrt2a* in regulation of hepatic LC-PUFA composition and lipid homeostasis. Zebrafish *dmrt2a* knockout via CRISPR/Cas9 causes functional loss and altered expression of genes governing LC-PUFA synthesis (*fads2*↓, *elovl8b*↑) and lipid metabolism (*rimkla*↓, *acot11*↑, *ptges3a*↑). These changes reshape the liver LC-PUFA composition (decreased EPA, DHA, and ARA), potentially contributing to lipid overload, indicated by elevated lipid droplet counts and triglyceride levels. A PUFA-enriched diet partially rescues this phenotype. LC-PUFA, long-chain polyunsaturated fatty acid; EPA, eicosapentaenoic acid; DHA, docosahexaenoic acid; ARA, arachidonic acid; WT, wild type.

## Data Availability

The RNA-seq data presented in the current study have been submitted to the NCBI BioProject database under accession number PRJNA1466040.

## Supplementary Materials

The following supporting information can be downloaded at https://www.mdpi.com/article/10.3390/cells15191763/s1, Table S1: Proportions of major components in the basal diet. Table S2: Fatty acid composition of the fish oil-supplemented diet. Table S3: Primers used in this study. Table S4: RNA sequencing summary statistics for livers from *dmrt2a*^−/−^ and wild-type fish. Table S5: Differentially expressed genes in livers of *dmrt2a*^−/−^ mutants compared with wild-type siblings (|log_2_FC| ≥ 1, adjusted *p* ≤ 0.05). Table S6: Fatty acid content in the wild-type and *dmrt2a* deletion fish livers (Mol%).

## Abbreviations

The following abbreviations are used in this manuscript:

LC-PUFA	long-chain polyunsaturated fatty acids
EPA	eicosapentaenoic acid
DHA	docosahexaenoic acid
ARA	arachidonic acid
DMRT	doublesex and mab-3 related transcription factor
gRNA	guide RNA
WT	wild-type
ORO	Oil Red O
Hetero	heterozygous
Homo	homozygous
RT-qPCR	real-time quantitative PCR
GO	Gene Ontology
KEGG	Kyoto Encyclopedia of Genes and Genomes
FAMEs	fatty acid methyl esters
SEM	standard error of the mean
FC	fold change
